# Killer-cell immunoglobulin-like receptors define a potent effector program in human **γδ** T cells

**DOI:** 10.1172/jci.insight.201160

**Published:** 2026-04-23

**Authors:** Mahya Razmi, Yeganeh Almasi, Marilee Larrivée, Jonathan B. Angel, Alexandre Blais, Zakia Djaoud

**Affiliations:** 1 Department of Biochemistry, Microbiology and Immunology, University of Ottawa, Ottawa, Ontario, Canada.; 2 Children’s Hospital of Eastern Ontario Research Institute, Ottawa, Ontario, Canada.; 3 University of Ottawa Centre for Inflammation, Immunity and Infection (CI3), Ottawa, Ontario, Canada.; 4 Inflammation and Chronic Disease Program, Ottawa Hospital Research Institute, Ottawa, Ontario, Canada.; 5 Ottawa Institute of Systems Biology, Ottawa, Ontario, Canada.; 6 Éric Poulin Centre for Neuromuscular Disease, Ottawa, Ontario, Canada.; 7 Ottawa Brain and Mind Research Institute, Ottawa, Ontario, Canada.

**Keywords:** Hematology, Immunology, Innate immunity, NK cells

## Abstract

Human γδ T cells are a rare but functionally diverse lymphocyte subset critical for tumor surveillance and antimicrobial immunity. Although they express NK cell–associated receptors such as killer-cell immunoglobulin-like receptors (KIRs), the relevance of KIR expression on γδ T cells remains largely unexplored. Using flow cytometry, ATAC-seq, and RNA-seq, we identified KIR expression as a marker that distinguished 2 functionally and molecularly distinct γδ T cell subsets. KIR^+^ γδ T cells exhibited an advanced, memory-like differentiation state characterized by heightened cytotoxicity, stable epigenetic remodeling, and a predominant IFN-γ–producing profile. In contrast, KIR^–^ γδ T cells maintained a naive-like phenotype and preferentially produced IL-17 upon polarization. Notably, KIR^+^ γδ T cells were consistently observed across individuals but were significantly enriched in cytomegalovirus (CMV)-seropositive donors, suggesting that chronic antigenic stimulation could promote the emergence of KIR^+^ effector γδ T cells. These findings reveal a functional dichotomy in human γδ T cells defined by KIR expression, linking IFN-γ–driven cytotoxicity with KIR^+^ cells and IL-17 production with KIR^–^ cells. This insight advances our understanding of γδ T cell heterogeneity and has implications for viral immunity, immune memory, and the development of γδ T cell–based immunotherapies.

## Abstract 2

Surface proteins called KIRs distinguish two human γδ T-cell subsets: one memory-like with strong effector potential, and another less-differentiated subset with a naïve-like phenotype.

## Graphical abstract

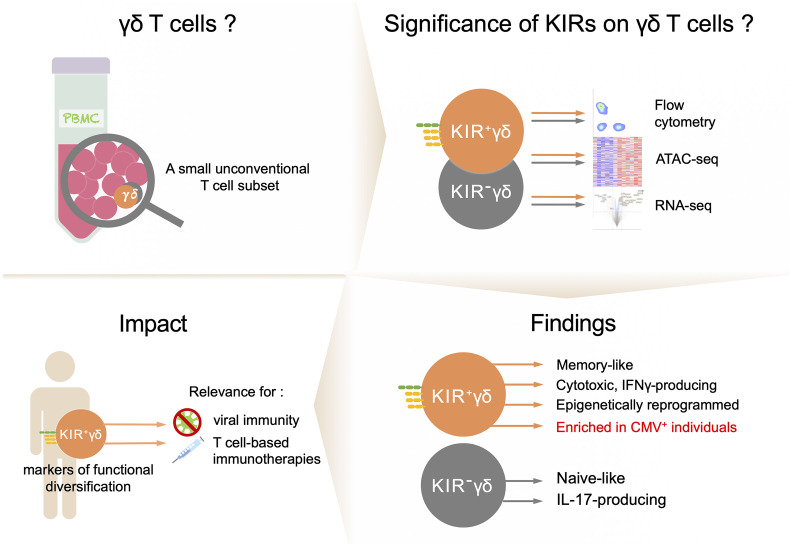

## Introduction

Unlike αβ T cells, most γδ T cells can recognize antigens independently of Human Leukocyte Antigen (HLA) class I presentation. This positions them as innate T cells, capable of detecting a wide array of structurally diverse ligands, allowing them to act as early responders to infection (1, 2). In peripheral blood, they typically account for less than 5% of total T cells, with the majority expressing the Vγ9Vδ2 T cell receptor (TCR). During certain bacterial and protozoan infections, however, Vγ9Vδ2 T cells can undergo massive expansion, sometimes reaching up to 50% of total T cells (3, 4). This expansion is driven by their ability to recognize small, phosphorylated metabolites known as phosphoantigens, which are produced by microbes via the isoprenoid biosynthesis pathway (5, 6).

In mice, γδ T cells undergo functional differentiation in the thymus into IL-17–producing or IFN-γ–producing subsets during distinct developmental stages (7). In humans, however, a comparable functional classification has not yet been firmly established. Most human γδ T cells exhibit cytotoxic activity and produce IFN-γ (8), while IL-17 production is minimal and appears to be induced only upon peripheral activation (9, 10). Consequently, human γδ T cells are broadly categorized into Vδ1, Vδ2, and Vδ1/2^–^ subsets based on TCR δ chain usage and the availability of specific antibodies for their detection. In peripheral blood, Vδ2 T cells predominate, whereas Vδ1 and Vδ1/2^–^ subsets are less frequent and more commonly enriched in tissues.

Recent studies have begun to uncover functionally distinct γδ T cell phenotypes in humans. In colorectal cancer tumors, 1 subset characterized by amphiregulin (AREG) production exhibits protumor properties, whereas another subset demonstrates cytotoxic antitumor activity (11). These findings suggest that AREG may serve as a more relevant functional marker for distinguishing γδ T cell subsets in the context of human cancer.

Although distinct from conventional T cells, γδ T cells — particularly the Vδ2^–^ subsets — can also exhibit features of adaptive immunity. A defining feature of adaptive responses is the ability of lymphocytes to undergo clonal expansion following antigen exposure and to generate memory cells that mediate faster and more effective responses upon reinfection. Certain γδ T cell subsets display these adaptive-like characteristics, expanding clonally and acquiring memory-like phenotypes in the context of infections such as HIV (12–14), cytomegalovirus (CMV) (15–19), *M. tuberculosis* (20, 21), and malaria (22–24). These adaptive-like T cell responses underscore their capacity for immunological memory, particularly in settings of chronic or repeated antigen exposure.

Supporting their clinical relevance, large-scale immunogenomic studies have identified γδ T cells as strong positive prognostic indicators across diverse cancer types (25, 26). However, their role in cancer is complex: while many γδ T cell subsets exhibit potent cytotoxicity, IL-17–producing γδ T cells have been implicated in tumor promotion (27, 28), likely through proinflammatory and proangiogenic effects (29, 30). This functional duality likely reflects heterogeneity in the γδ T cell subsets recruited to the tumor and their plasticity in response to cues from the tumor microenvironment.

A recent study provided additional nuance, demonstrating that γδ T cells infiltrating mismatch repair-deficient colorectal cancers, particularly those expressing killer-cell immunoglobulin-like receptors (KIRs), are associated with improved patient outcomes (31). KIRs, traditionally studied in the context of NK cell education through interactions with HLA class I molecules (32), remain poorly understood in γδ T cells. Notably, persistent viral infections such as CMV are known to drive lymphocyte maturation. In NK cells, CMV infection induces the expansion of a differentiated, adaptive-like subset characterized by the expression of both KIR and the activating receptor NKG2C (33–36). Given the shared expression of NK cell receptors by γδ T cells, we investigated the expression of KIRs within the human γδ T cell compartment, its relationship to CMV serostatus, and whether coexpression with NKG2C delineates a mature, memory-like γδ T cell population. Flow cytometry analysis, ATAC-seq and RNA-seq reveal that KIR expression on human γδ T cells defines a distinct subset with enhanced effector capacity and phenotypic features of adaptation, independent of NKG2C. These findings position KIR^+^ γδ T cells as a promising adaptive-like population with potential utility in adoptive immunotherapy.

## Results

### CMV seropositivity is associated with KIR expression on γδ T cells.

CMV drives a gradual expansion of epitope-specific T cell subsets over time in healthy individuals (37, 38). In CMV-seropositive (CMV^+^) individuals, NK cells acquire a distinct phenotype marked by an increased frequency of circulating CD56^dim^NK cells (39) and a highly differentiated, memory-like CD57^+^NKG2C^+^KIR^+^ profile (33–36). A similar adaptation is observed in the γδ T cell compartment, where CMV^+^ individuals display a shift toward an effector memory phenotype, including a higher proportion of Vδ1 T cells compared with CMV-seronegative (CMV^–^) individuals (15–19). Given the shared expression of NK cell markers by γδ T cells and the phenotypic parallels between these 2 compartments in the context of CMV, we examined whether CMV similarly influences the frequency of NKG2C^+^KIR^+^ γδ T cells by comparing CMV^+^ and CMV^–^ individuals.

**Figure 1:**
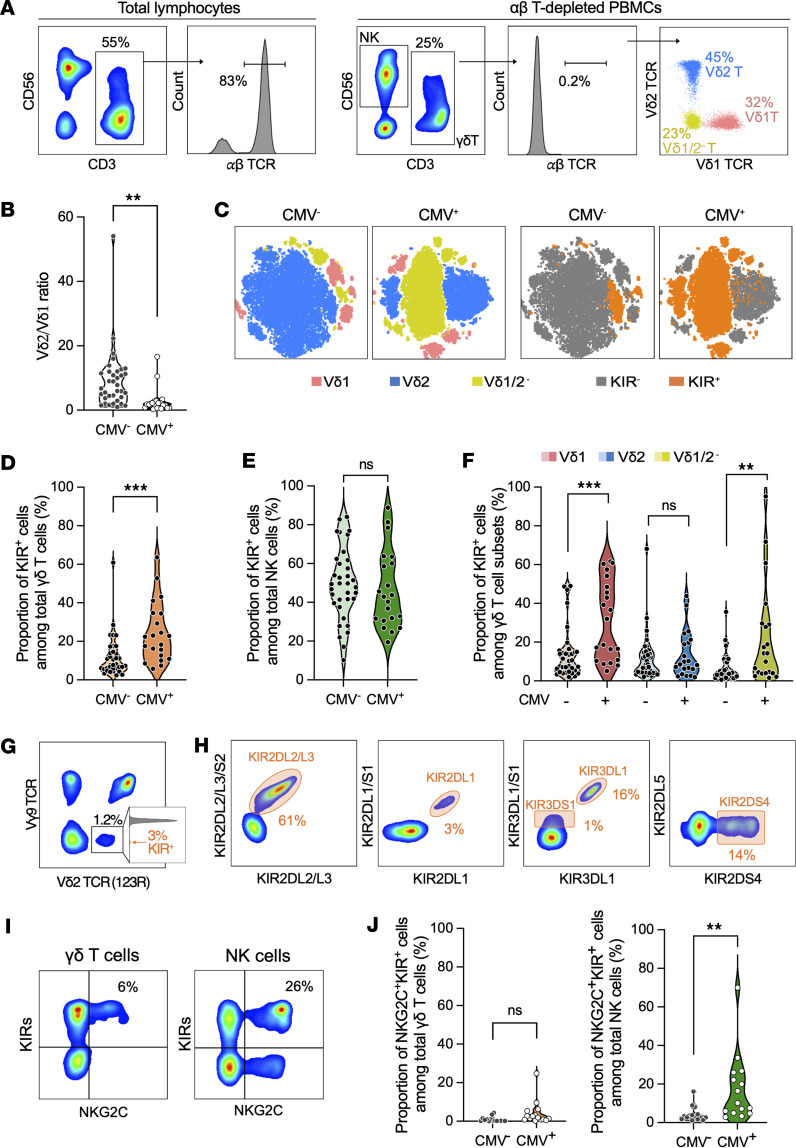
CMV-serostatus influences KIR expression by γδ T cells. (**A**) Representative pseudocolor plots show the frequencies of CD3^+^ and αβ TCR^+^ cells within PBMCs (left panel) and within αβ T cell–depleted PBMCs (right panel). Vδ1, Vδ2, and Vδ1/2^–^ subsets are shown in the right panel, within αβ T cell–depleted PBMCs. (**B**) Violin plots depict Vδ2/Vδ1 ratios within γδ T cells from CMV^–^ and CMV^+^ individuals. Each dot represents a donor. (**C**) Representative t-SNE plots show the distribution of Vδ1, Vδ2, and Vδ1/2^–^ subsets (left), and of KIR^–^ and KIR^+^ cells (right), among γδ T cells in 2 representative CMV^–^ and CMV^+^ individuals. Vδ1, Vδ2, KIRs, CD56, and CD57 were used as input parameters to generate the t-SNE plots. Vδ1, Vδ2, and Vδ1/2^–^ populations were manually overlaid on the left panel, while KIR expression was overlaid on the right panel. (**D**–**F**) Violin plots show the proportions of KIR^+^ cells among γδ T cells (**D**), NK cells (**E**), or γδ T cell subsets (**F**). (**G**) A representative pseudocolor plot and histogram show the proportion of Vγ9^–^Vδ2^+^ cells within the γδ T cell population and the proportion of KIR^+^ cells within this subset. (**H**) Representative pseudocolor plots illustrate the expression of selected KIRs on γδ T cells, including KIR2DL2/L3^+^, KIR2DL1^+^, KIR3DS1^+^, KIR3DL1^+^, and KIR2DS4^+^ populations. (**I** and **J**) Representative pseudocolor plots (**I**) and corresponding violin plots (**J**) compare the frequencies of KIR^+^NKG2C^+^ γδ T cells (orange) and KIR^+^NKG2C^+^ NK cells (green) in CMV^–^ and CMV^+^ individuals. Each dot represents an individual donor. The flow cytometry analysis was performed on samples from 35 CMV^–^ and 21 CMV^+^ individuals (**A**–**E**) and 15 CMV^–^ and 15 CMV^+^ individuals (**J**). Statistical significance of the difference between CMV^–^ and CMV^+^ individuals was assessed using the unpaired 2-tailed *t* test (**P < 0.005, ***P < 0.0008).

Due to their relatively low frequency, comprising only ~0.5%–5% of total peripheral blood mononuclear cells (PBMCs), γδ T cells are challenging to study. However, through the targeted depletion of αβ T cells, the proportion of γδ T cells can be dramatically enhanced to ~10%–45% (Figure 1A). This enrichment facilitates more robust flow cytometry analyses of γδ T cell subsets and allows for direct phenotypic comparison with NK cells, which remain present in the same cultures (Figure 1A). To further investigate this, we immunophenotyped αβ T cell–depleted (αβ T-depleted) PBMCs from 34 CMV^–^ and 19 CMV^+^ healthy adult blood donors, including both males and females, and representing a range of ages and ethnic backgrounds. Although the age and sex differences between the 2 groups were not statistically significant, CMV prevalence was, as expected, lower among individuals of European origin compared with those from less industrialized regions with higher known seropositivity rates (40) (Supplemental Figure 1; supplemental material available online with this article; https://doi.org/10.1172/jci.insight.201160DS1).

Consistent with previous studies (15–19), CMV^+^ individuals showed a marked increase in Vδ1 T cell frequencies compared with CMV^–^ individuals, who predominantly exhibited Vδ2 T cells. Consequently, the Vδ2/Vδ1 ratio was markedly reduced in the CMV^+^ group (Figure 1B). CMV^+^ individuals also displayed higher frequencies of the Vδ1/2^–^ subset (Figure 1C). Moreover, we found that CMV^+^ individuals exhibit significantly higher proportions of KIR^+^ γδ T cells (Figure 1, C and D), whereas the proportion of KIR^+^ NK cells remained unaffected by CMV serostatus (Figure 1E). A closer examination of the γδ T cell subsets revealed that this phenotypic shift was primarily driven by the Vδ1 and Vδ1/2^–^ populations, while Vδ2 T cells remained unchanged (Figure 1F).

Davey et al. reported clonal expansion of Vγ9^–^Vδ2^+^ T cells during acute CMV infection (41). Our cohort comprises healthy individuals without active CMV infection, a setting in which the Vδ2^+^ compartment is largely dominated by Vγ9^+^Vδ2^+^ T cells. Accordingly, Vγ9^–^Vδ2^+^ T cells are either absent or present at very low frequencies. Specifically, this subset is absent in most CMV^–^ individuals and represents, on average, only ~1.5% of total Vδ2^+^ T cells in CMV^+^ individuals, with minimal KIR expression (Figure 1G). Notably, only 1 CMV^+^ individual shows a marked expansion of Vγ9^–^Vδ2^+^ T cells (~10%), of which ~30% are KIR^+^. Among the remaining individuals, 2 display intermediate frequencies (~4%), whereas all others range between 0.1% and 3% and exhibit little to no KIR expression (Supplemental Figure 2).

Depending on each donor’s KIR gene repertoire, γδ T cells expressed both activating and inhibitory KIRs, with inhibitory KIRs being more predominant. Among these, KIR2DL2/L3 was the most highly expressed (Figure 1H). Notably, unlike NK cells from CMV^+^ individuals, which typically show high levels of KIR and NKG2C coexpression (33–36), γδ T cells did not exhibit elevated NKG2C expression, and the KIR^+^NKG2C^+^ subset was not prominent in either CMV^–^ or CMV^+^ individuals (Figure 1, I and J).

Overall, these findings confirm previous reports of CMV-associated expansion of Vδ2^–^ γδ T cells (15–19) and further show that CMV seropositivity is associated with a selective enrichment of the KIR^+^ subset within the γδ T cell compartment. While CMV-driven expansion in NK cells is marked by upregulation of both NKG2C and KIRs (33–36), the corresponding imprint in γδ T cells appears to be predominantly characterized by increased KIR expression. This implicates KIR^+^γδ T cells in CMV immune surveillance and suggests a role for CMV in driving γδ T cell differentiation.

### KIRs define 2 distinct phenotypic signatures within γδ T cells.

**Figure 2:**
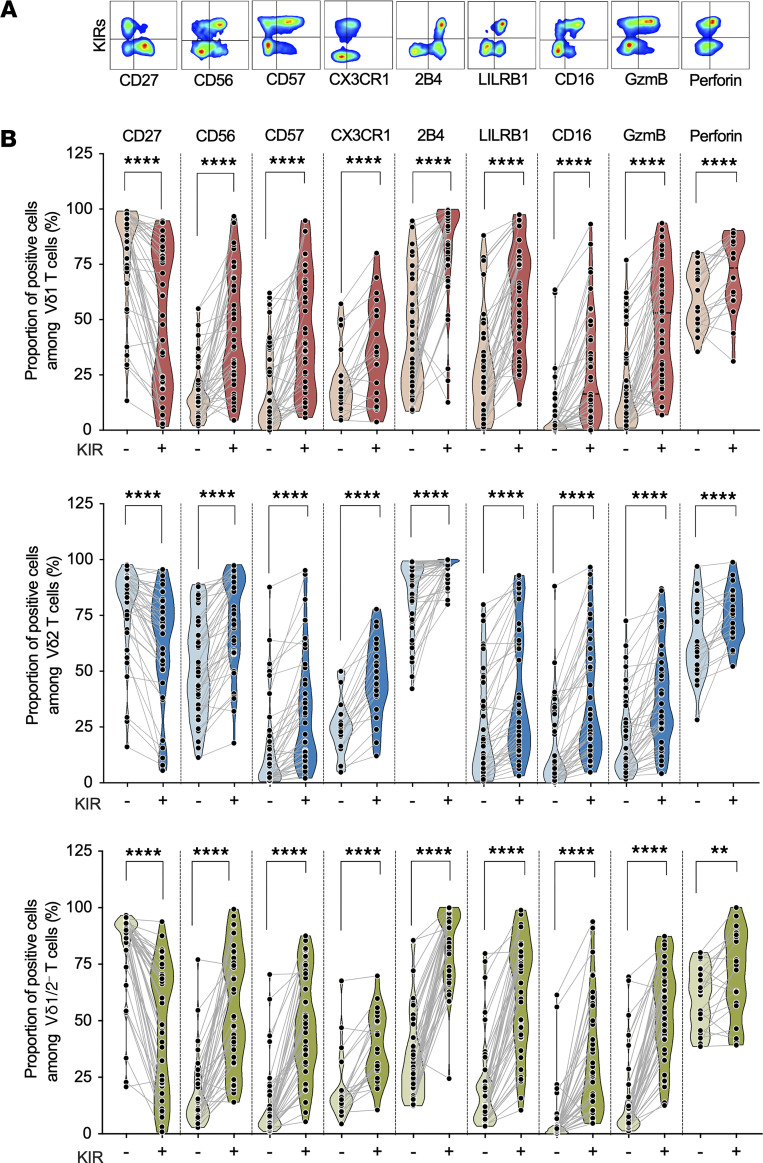
KIR^+^γδ T cells display enhanced maturity and cytotoxic potential compared with KIR^–^ γδ T cells. (**A**) Representative pseudocolor plots show the expression of the indicated markers by total γδ T cells. (**B**) Violin plots compare the proportions of KIR^–^ and KIR^+^ cells expressing the indicated markers within Vδ1 T cells (top panel), Vδ2 T cells (middle panel), and Vδ1/2^–^ T cells (bottom panel). Each dot represents an individual donor. The flow cytometry analysis was performed on samples from 41 individuals, except for CX3CR1 and Perforin, which were assessed in 21 and 20 individuals, respectively. Statistical significance of the difference between KIR^–^ and KIR^+^ cells was assessed using the paired 2-tailed *t* test (**P = 0.002, ****P < 0.0008).

We investigated whether KIR expression marks a more mature stage of γδ T cell differentiation. To this end, we compared the phenotype of KIR^–^ and KIR^+^ γδ T cells by analyzing the expression of selected surface and intracellular markers, using spectral flow cytometry. Our analysis demonstrated that KIR^+^ γδ T cells predominantly display a CD27^–^CD57^+^CX3CR1^+^ phenotype in both CMV^–^ and CMV^+^ individuals, consistent with advanced maturation and a memory-like profile. In contrast, KIR^–^ γδ T cells exhibit a CD27^+^CD57^–^CX3CR1^–^ phenotype, indicative of a less differentiated state. KIR^+^ γδ T cells also show elevated expression of CX3CR1 and NK cell–associated receptors, including CD56, 2B4, LILRB1, and CD16. Additionally, they contain high levels of granzyme B and perforin, key effector molecules involved in cytotoxic activity (Figure 2A). Notably, NKG2A and NKG2C are consistently expressed at higher levels on KIR^+^ Vδ1 T cells compared with their KIR^–^ counterparts; however, this pattern is not observed in the Vδ2 subset, and within the Vδ1/2^–^ subset, only NKG2C is upregulated on KIR^+^ cells (Supplemental Figure 3A). In contrast, NKG2D expression remains comparable between KIR^–^ and KIR^+^ cells across all subsets analyzed (Supplemental Figure 3B). We further observed that exhaustion markers such as LAG-3, CTLA-4, and TIM-3 are minimally expressed on γδ T cells, regardless of KIR status. While PD-1 is present, its expression is not enriched within the KIR^+^ population. TIGIT expression is notably higher on KIR^+^ γδ T cells relative to KIR^–^ cells, aligning with a memory-like effector phenotype rather than exhaustion (Supplemental Figure 3C). This is consistent with TIGIT’s enrichment on effector memory T cells compared with naive cells and its association with enhanced cytotoxic potential (42, 43).

**Figure 3:**
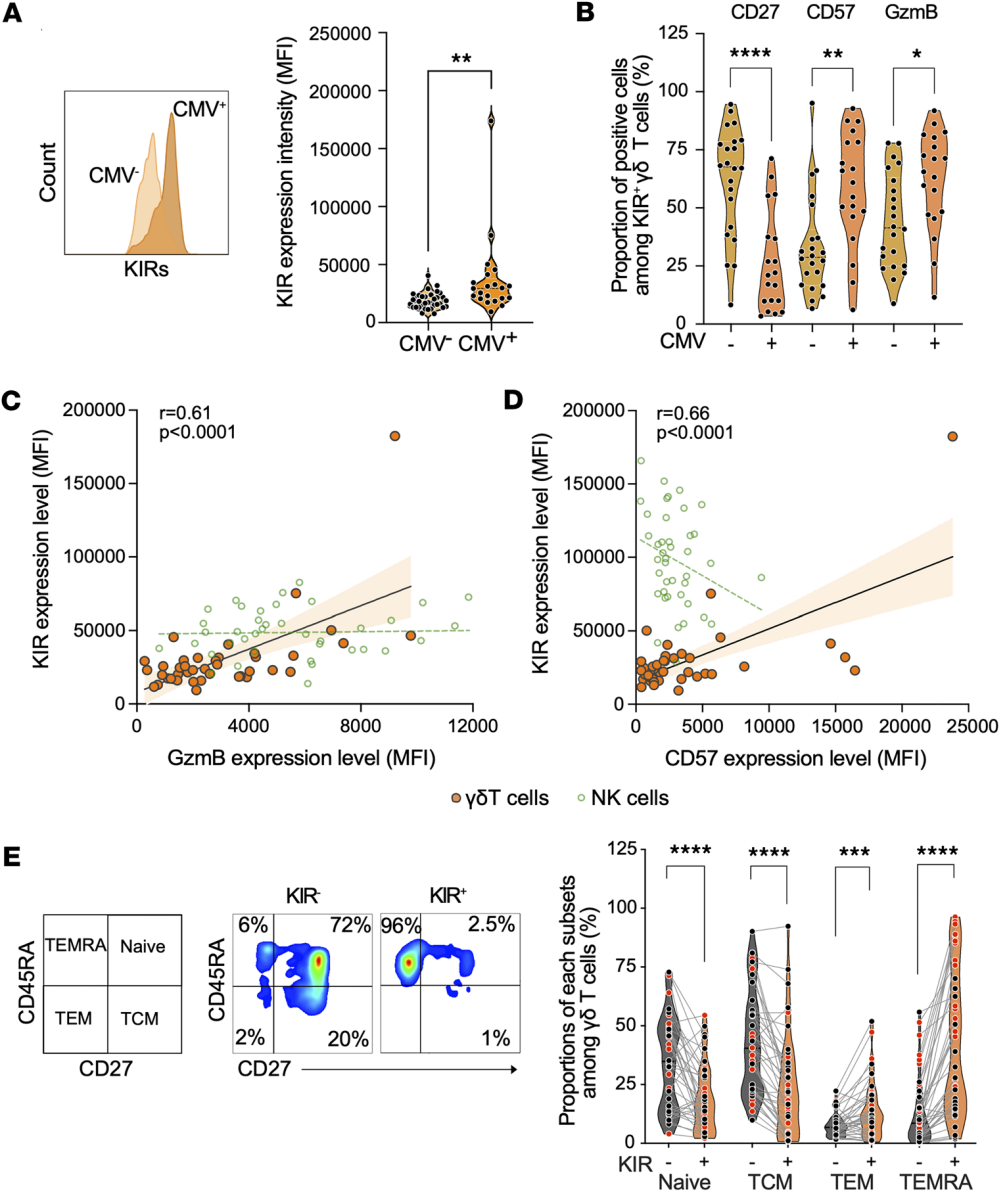
KIR^+^ γδ T cells have a TEMRA phenotype. (**A**) Representative histogram overlays (left panel) and corresponding violin plots (right panel) show KIR expression intensity by total γδ T cells from CMV^−^ and CMV^+^ individuals (*n* = 56; 35 CMV^–^, 21 CMV^+^). (**B**) Violin plots show the proportions of KIR^+^ γδ T cells expressing CD27, CD57, and granzyme B in CMV^–^ and CMV^+^ individuals. Each dot represents an individual donor (*n* = 56; 22 CMV^–^, 19 CMV^+^). Statistical significance of the difference between KIR^–^ and KIR^+^ cells was assessed using the unpaired 2-tailed *t* test (*P = 0.01, **P ≤ 0.001, ****P < 0.0001). (**C** and **D**) Correlation between KIR and granzyme B (**C**), and KIR and CD57 (**D**) expression intensities in γδ T cells (orange) and NK cells (green). Each dot represents an individual donor (*n* = 41). Expression intensities were measured by flow cytometry. A simple linear regression fit line is shown, and Pearson correlation analysis was used to assess the associations between the indicated parameters. (**E**) Representative pseudocolor plots (left panel) and the corresponding violin plots (right panel) show the distribution of CD27/CD45RA-defined subsets among KIR^–^ and KIR^+^ γδ T cells, including naive, central memory (TCM), effector memory (TEM), and terminally differentiated effector memory (TEMRA) populations. Each dot represents an individual donor (*n* = 40). Samples from CMV^+^ individuals are shown in red symbols. Statistical significance of the difference between KIR^–^ and KIR^+^ cells was assessed using the paired 2-tailed *t* test (***P = 0.0004, ****P < 0.0001).

The observed phenotypic distinction between KIR^–^ and KIR^+^ cells was most evident within the Vδ2^–^ subsets but was consistently observed across Vδ1, Vδ2, and Vδ1/2^–^ γδ T cell populations when stratified by KIR expression (Figure 2B). Importantly, when these subsets were further analyzed according to CMV serostatus, a clear gradation in differentiation emerged across the 4 major groups (KIR^–^CMV^–^, KIR^–^CMV^+^, KIR^+^CMV^–^, and KIR^+^CMV^+^). Indeed, while the KIR-associated phenotypic dichotomy was detectable in both CMV^–^ and CMV^+^ individuals, it was markedly amplified in the latter. γδ T cells from CMV^+^ donors exhibited higher KIR expression intensity (Figure 3A) and, in parallel, a more advanced differentiation profile characterized by further downregulation of CD27, increased CD57 expression, and enhanced granzyme B content (Figure 3B). Together, these data highlight a progressive phenotypic continuum across KIR-defined subsets shaped by CMV serostatus, with the most differentiated phenotype observed in KIR^+^ γδ T cells from CMV^+^ individuals.

We next assessed the correlation between the expression intensity of KIRs and that of granzyme B and CD57, the latter being a marker of advanced differentiation and cytotoxic potential in NK cells (33, 44, 45). In γδ T cells, higher KIR expression intensity was associated with increased detection of granzyme B and CD57 (Figure 3, C and D). These associations were modest but statistically supported and were consistently observed across donors. Notably, comparable associations were not detected in NK cells analyzed in parallel (Figure 3, C and D). This distinction is of interest given that, in NK cells, KIR expression in combination with NKG2C defines a mature and highly cytotoxic subset characterized by elevated CD57 and granzyme B expression (33, 34, 44, 45). Together, these observations indicate that, in γδ T cells, KIR expression is associated with differentiation and cytotoxic features through mechanisms that differ from the canonical NKG2C-associated maturation pathway described in NK cells.

To further characterize the differentiation status of γδ T cells according to their expression of KIRs, we assessed the expression of CD27 and CD45RA to distinguish naive (CD45RA^+^CD27^+^), central memory (TCM; CD45RA^–^CD27^+^), effector memory (TEM; CD45RA^–^CD27^–^), and terminally differentiated effector memory (TEMRA; CD45RA^+^CD27^–^) subsets. We found that the majority of KIR^+^ γδ T cells exhibit a TEMRA phenotype, whereas KIR^–^ γδ T cells are predominantly naive or display a TCM phenotype (Figure 3E). This phenotypic pattern is consistently observed across all Vδ1, Vδ2, and Vδ1/2^–^ subsets (Supplemental Figure 4).

In conclusion, KIR expression identifies a highly differentiated cytotoxic subset of γδ T cells, particularly enriched in CMV^+^ individuals. These KIR^+^ γδ T cells exhibit a TEMRA phenotype and express high levels of CD57, CX3CR1, NK cell receptors, and cytotoxic molecules. However, unlike NK cells, where KIR and NKG2C coexpression defines a mature cytotoxic subset, γδ T cells show KIR-associated maturation independent of NKG2C. This highlights a distinct, adaptive-like differentiation pathway in γδ T cells, positioning KIRs as key markers of their functional maturation.

### KIR-associated dichotomy revealed at the epigenetic and transcriptomic levels.

**Figure 4:**
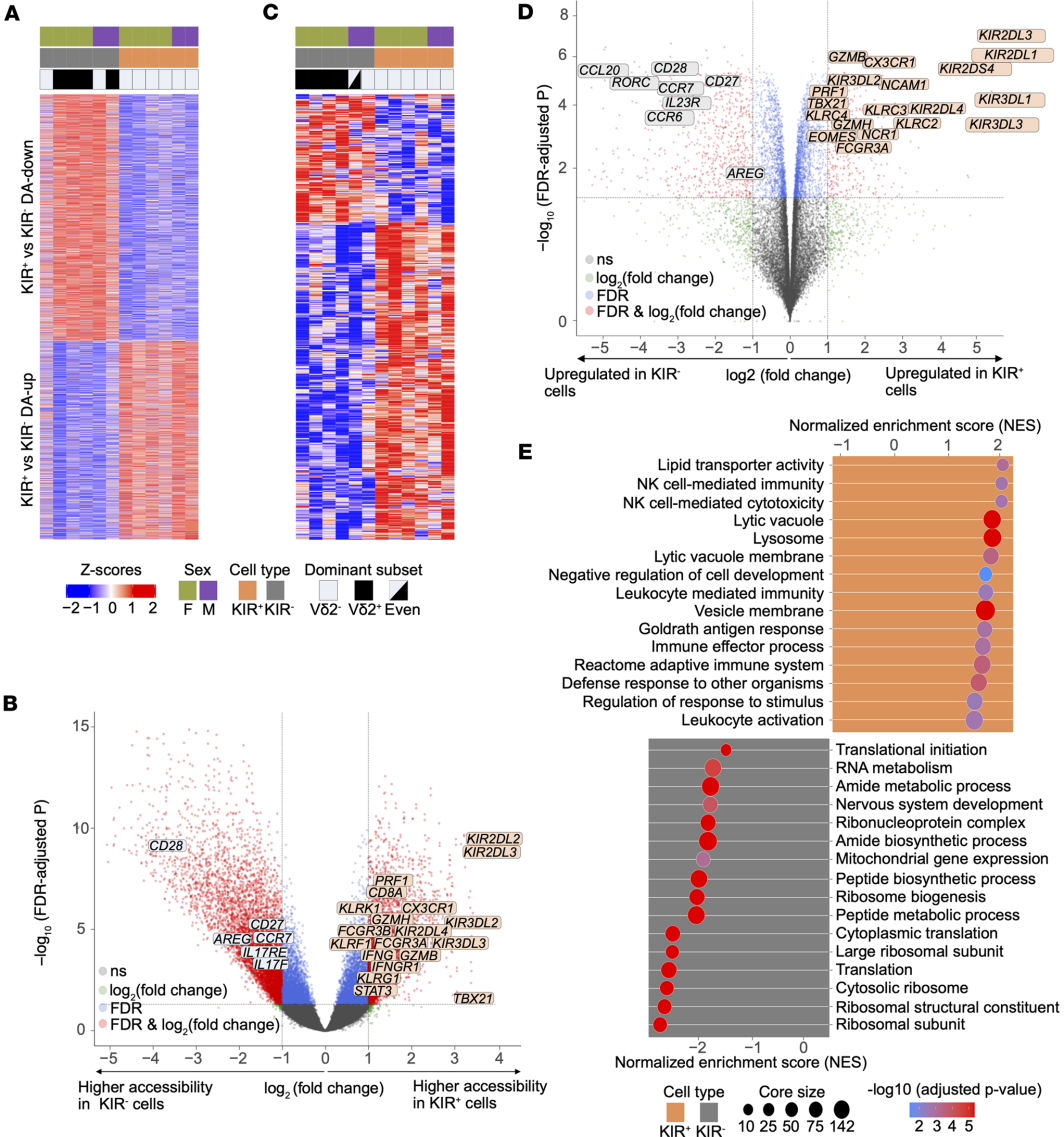
Epigenetic and transcriptomic profiling of γδ T cells reveals a KIR associated divergence. (**A**) Heatmap of differentially accessible regions (DARs) identified by ATAC-seq between KIR^+^ and KIR^–^ γδ T cells. Sex, cell type (KIR^–^ versus KIR^+^), and dominant subsets are provided. Rows (regions of differential accessibility) were ordered by hierarchical clustering. (**B**) Volcano plot of differential DNA accessibility in KIR^+^ and KIR^–^ γδ T cells. Genomic windows with significantly different accessibility and located near genes of interest are labeled with the symbol and colored based on the γδ T cell subset showing higher accessibility. (**C**) Heatmap of gene expression differences between KIR^+^ and KIR^–^ γδ T cells, organized as in **A**. (**D**) Volcano plot of differences in gene expression between KIR^+^ and KIR^–^ γδ T cells. Select genes are labeled and colored by the direction of expression difference. (**E**) Results of gene set enrichment analysis using MSigDB Gene Ontology, Reactome and “Hallmark” gene sets. Only selected sets are shown. Node size is proportional to the number of genes contributing to the “core enrichment” of each set, while the adjusted *P* value (*P* < 0.05) is encoded by color. Samples from 6 individuals were used for both ATAC-seq and RNA-seq analysis.

To further investigate the molecular basis of the KIR-associated phenotypic signature, we performed ATAC-seq as previously described (46, 47). Chromatin accessibility was analyzed in γδ T cells isolated from the blood of 6 CMV^+^ donors, 4 of whom were also included in bulk RNA-seq analysis. For both ATAC-seq and RNA-seq, analyses were performed on 50,000 sorted KIR^–^ and KIR^+^ γδ T cells per sample to ensure sufficient coverage. Donors were selected based on sufficient representation of KIR^+^ γδ T cells to ensure robust profiling of both subsets. In all donors, the analysis identified approximately 2,000 differentially accessible regions between KIR^–^ and KIR^+^ γδ T cells (Figure 4A), with chromatin accessibility patterns closely aligning with the flow cytometry data. No differences were observed based on the sex of the donors, indicating that the epigenetic landscape distinguishing KIR^–^ and KIR^+^ γδ T cells is consistent across male and female individuals. Differential accessibility within KIR genes was observed for *KIR2DL2*, *KIR2DL3*, *KIR2DL4*, *KIR3DL2*, and *KIR3DL3*. Notably, KIR^+^ γδ T cells exhibited increased chromatin accessibility at key effector loci, including genes encoding NK cell–associated receptors, such as *KLRF1* (NKp80), *KLRG1*, *KLRK1* (NKG2D), and *FCGR3* (CD16), as well as genes involved in cytotoxic functions and effector differentiation, including *IFNG*, *IFNGR1*, *GZMB*, *GZMH*, *PRF1*, *TBX21* (T-bet), *STAT3*, and *CX3CR1*. In contrast, KIR^–^ γδ T cells exhibited greater chromatin accessibility at loci associated with naive T cell markers such as *CD27*, *CD28*, and *CCR7*, as well as genes linked to IL-17 biology. These included *IL17F* and *IL17RE*, which are implicated in protumorigenic inflammation (27). Accessibility was also increased at *AREG*, which encodes AREG, a tissue-repair factor secreted by IL-17–producing γδ T cells (11) (Figure 4B).

Transcriptomic analyses also conducted on samples from 6 CMV^+^ donors further revealed distinct gene expression profiles between KIR^–^ and KIR^+^ γδ T cells (Figure 4C). KIR^+^ cells exhibited upregulation of cytotoxicity-related genes such as *GZMB*, *GZMH*, *PRF1*, *CX3CR1*, and *TBX21*, as well as NK cell–associated genes including *NCR*1 (NKp46), *KLRC2* (NKG2C), *KLRC3* (NKG2E), *KLRC4* (NKG2F), and *NCAM1* (CD56). Multiple KIR genes, including *KIR2DL1*, *KIR2DL2*, *KIR2DL3*, *KIR2DL4*, *KIR2DS4*, *KIR3DL1*, *KIR3DL2*, and *KIR3DL3*, were also upregulated in the KIR^+^ subset (Figure 4D). In line with the epigenetic findings, *CD27*, *CCR7*, and *CD28* were more highly expressed in KIR^–^ γδ T cells. This subset also exhibited upregulation of genes characteristic of IL-17–producing γδ T cells, including *RORC* (encoding the IL-17–lineage-defining transcription factor RORγt) (48, 49), *AREG*, *IL23R* (required for IL-17 polarization) (9), and the chemokine receptor-ligand pair *CCR6* and *CCL20*, which are essential for trafficking IL-17–producing γδ T cells to inflamed tissues such as skin and liver (10, 50, 51) (Figure 4D).

Given that the majority of KIR^+^ γδ T cells were Vδ2^–^ in many individuals of our cohort (Figure 1F), we considered whether the observed transcriptional and epigenetic dichotomy might simply reflect the distribution of Vδ2^+^ and Vδ2^–^ cells between KIR^+^ and KIR^–^ subsets. However, both ATAC-seq and RNA-seq analyses included samples in which KIR^–^ γδ T cells contained a substantial proportion of Vδ2^–^ cells (Figure 4, A and C). This supports the conclusion that the observed molecular differences are indeed associated with KIR expression status rather than TCRδ chain usage.

To identify gene sets that were enriched either in KIR^+^ or KIR^–^ γδ T cells, we conducted gene set enrichment analysis (GSEA) (52, 53). In KIR^+^ γδ T cells, we observed significant enrichment of gene sets associated with immune activation, effector function, and adaptive immunity. Specifically, gene sets linked to leukocyte activation, immune effector processes, NK cell–mediated cytotoxicity, and antigen response were all enriched, indicating that these cells are transcriptionally primed for rapid and robust immune responses. Additionally, elevated expression of genes involved in vesicle-mediated transport, lysosomal function, and lipid transporter activity further supports a transcriptional profile consistent with cytotoxicity and intercellular communication, all of which are hallmarks of an effector-like immune state (Figure 4E). In contrast, KIR^–^ γδ T cells showed enrichment for gene sets linked to ribosome biogenesis, cytoplasmic translation, RNA metabolism, and mitochondrial gene expression. This enrichment pattern indicates a regulatory landscape geared toward biosynthetic and translational processes, suggesting that these cells are transcriptionally poised for protein production and cellular growth rather than immediate effector function (Figure 4E).

Collectively, these results reveal a previously unrecognized epigenetic and transcriptional bifurcation within human γδ T cells defined by KIR expression, with KIR^+^ cells exhibiting features of highly functional effector cells, and KIR^–^ cells displaying a gene expression program suggestive of a more naive, proliferative, or IL-17–biased phenotype.

### Functional divergence of γδ T cells with IFN-γ–producing KIR^+^ cells versus IL-17–producing KIR^–^ cells.

**Figure 5:**
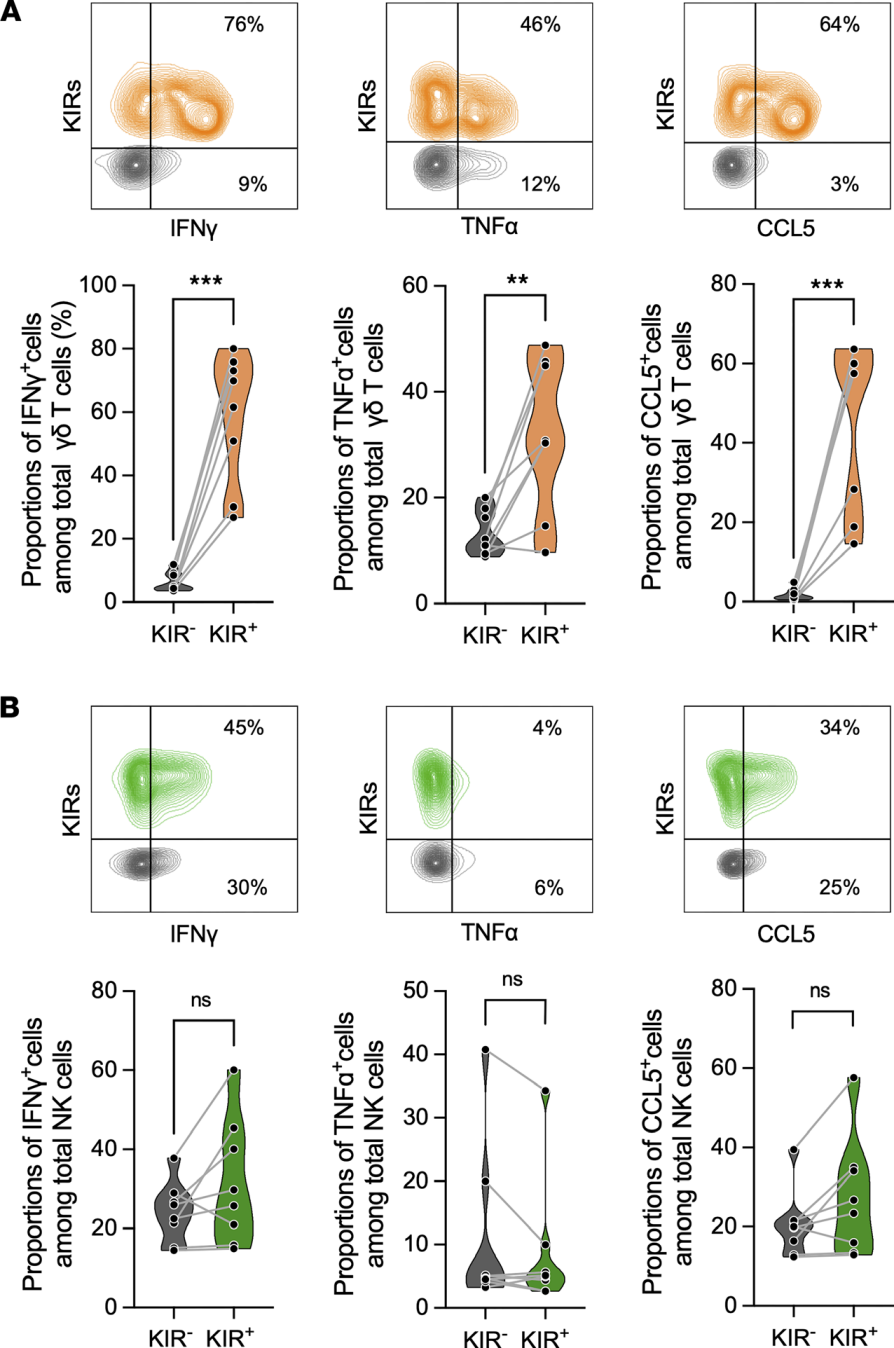
KIR^+^ γδ T cells exhibit elevated levels of cytokine production following activation. (**A**) Representative overlaid contour plots (top panel) and corresponding violin plots (bottom panel) compare the proportions of KIR^–^ (grey) and KIR^+^ (orange) γδ T cells producing IFN-γ, TNF-α, and CCL5, after 4 hours of stimulation with PMA/ionomycin. (**B**) Representative overlaid contour plots (top panel) and corresponding violin plots (bottom panel) compare the proportions of KIR^–^ (gray) and KIR^+^ (green) NK cells producing IFN-γ, TNF-α, and CCL5 under the same stimulation conditions. Each dot represents an individual donor. The flow cytometry analysis was performed on samples from 4 CMV^–^ and 4 CMV^+^ individuals. Statistical significance of the difference between KIR^–^ and KIR^+^ subsets was assessed using the paired 2-tailed *t* test (**P < 0.005, ***P < 0.0001).

While we have shown that KIR^+^ γδ T cells contain higher levels of cytotoxic granules (Figure 2), it remained unclear whether the 2 subsets differ in their cytokine responses to stimulation. ATAC-seq revealed greater chromatin accessibility at the *IFNG* locus in KIR^+^ γδ T cells, and transcriptomic analysis showed higher expression of this gene in the same subset. However, both analyses were conducted on freshly sorted resting cells. To determine whether these distinct epigenetic and transcriptomic profiles translate into functional differences under stimulatory conditions, we stimulated αβ T cell–depleted PBMCs with PMA/ionomycin for 4 hours, a short time frame designed to capture early activation events. We then assessed intracellular production of IFN-γ, TNF-α, and CCL5 in KIR^–^ and KIR^+^ γδ T cell subsets, with NK cells included for comparison. Within this brief stimulation window, KIR^+^ γδ T cells produced significantly higher levels of all 3 cytokines compared with their KIR^–^ counterparts (Figure 5A), indicating a rapid and heightened response. In contrast, no significant differences in cytokine production were observed between KIR^–^ and KIR^+^ NK cells (Figure 5B). This differential capacity to produce IFN-γ, TNF-α, and CCL5 between KIR^–^ and KIR^+^ γδ T cells was consistently observed across Vδ1, Vδ2, and Vδ1/2^–^ γδ T cell subsets (Supplemental Figure 5).

When evaluating antibody-dependent cellular cytotoxicity (ADCC) against Rituximab-coated Raji cells, Vδ1 and Vδ1/2^–^ γδ T cell subsets displayed comparable cytotoxic responses regardless of KIR expression. In contrast, within the Vδ2 γδ T cell subset, KIR expression was consistently associated with a marked increase in ADCC, accompanied by enhanced IFN-γ production (Supplemental Figure 6A). This selective enhancement observed in KIR^+^ Vδ2 γδ T cells could not be explained by differences in CD16 expression intensity among the Vδ1, Vδ2, and Vδ1/2^–^ γδ T cell subsets (Supplemental Figure 6B). These findings indicate that the functional association between KIR expression and ADCC is not uniform across γδ T cell subsets but is selectively evident within the Vδ2 γδ T cell compartment.

**Figure 6:**
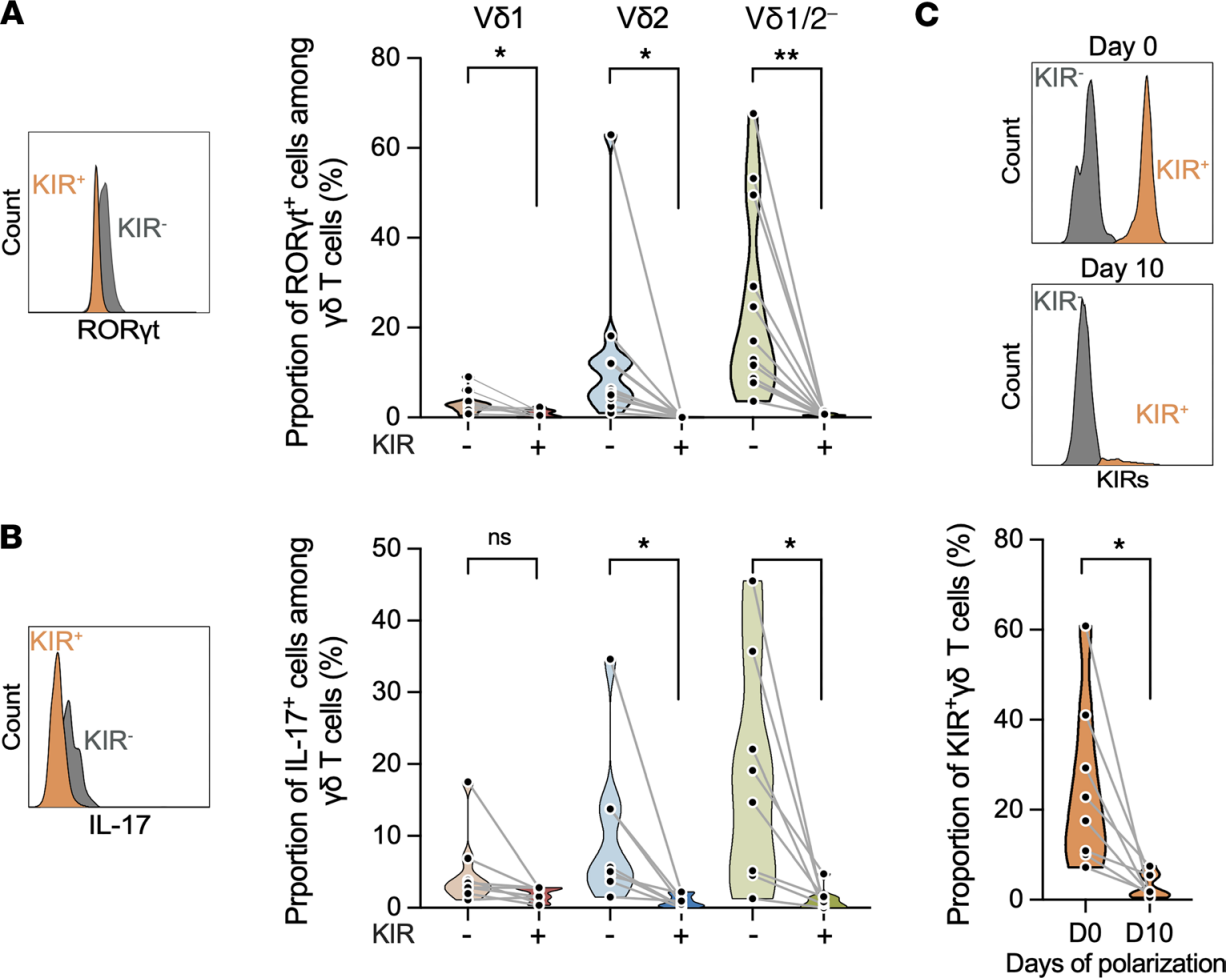
KIR^–^ γδ T cells show increased RORγt and IL-17 expression after polarization. (**A**–**C**) αβ T cell–depleted PBMCs were cultured for 4 days with IL-1β, IL-6, IL-23, and TGF-β (**A** and **B**) or for 10 days in the same cytokine cocktail supplemented with irradiated allogeneic PBMC feeder cells (**C**). (**A** and **B**) Representative overlaid histograms (left panels) and corresponding violin plots (right panels) compare RORγt and IL-17 expression between KIR^–^ and KIR^+^ γδ T cells after 4 days of polarization. (**C**) Representative overlaid histograms (top panels) and corresponding violin plots (bottom panel) show KIR expression at Day 0 and Day 10 of polarization. Each dot represents an individual donor (*n* = 12, **A**; *n* = 8, **B** and **C**). Statistical significance of the difference between KIR^–^ and KIR^+^ subsets was assessed using the paired 2-tailed *t* test (*P < 0.05, **P < 0.005).

We next investigated whether the epigenetic data indicating that IL-17–producing γδ T cells reside predominantly within the KIR^–^ subset could be confirmed functionally. To do this, we polarized γδ T cells for 4 days in the presence of IL-23, IL-6, IL-1β, and TGF-β, followed by activation with PMA/ionomycin. The results show a clear upregulation of RORγt in all KIR^–^ subsets (Figure 6A), which consistently displayed a higher proportion of IL-17–producing cells than their KIR^+^ counterparts. Of note, while a significantly higher fraction of KIR^–^ cells produced detectable IL-17 compared with their KIR^+^ counterparts across γδ T cell subsets, the overall level of IL-17 expression remained modest (Figure 6B). Moreover, extended culture for 10 days under these IL-17–polarizing conditions led to a progressive loss of KIR expression in γδ T cells (Figure 6C), suggesting that KIR^–^ γδ T cells are preferentially maintained in this environment.

In conclusion, our data establish KIR expression as a robust marker linked to enhanced effector functions in γδ T cells. The elevated production of IFN-γ, TNF-α, and CCL5 following stimulation across all γδ T cell subsets aligns with the distinct epigenetic and transcriptomic signatures of KIR^+^ γδ T cells. Conversely, the preferential polarization and maintenance of IL-17–producing γδ T cells within the KIR^–^ compartment further emphasize the functional divergence marked by KIR expression, underscoring the complexity of γδ T cell specialization.

## Discussion

Our study demonstrates that KIR expression marks a functionally distinct subset of human γδ T cells with a TEMRA-like phenotype, characterized by rapid cytokine responses, a highly differentiated phenotype and features consistent with memory-like functionality. Previous studies have shown that subsets of Vδ1 T cells can undergo antigen-driven differentiation and adaptive transcriptional reprogramming, giving rise to potent effector cells (18, 19, 54). However, identifying these differentiated populations is challenging, and it remains unclear whether similar differentiation occurs across other γδ T cell subsets. Here, we show that KIR expression is consistently associated with a highly differentiated functional state across γδ T cells, independent of TCR usage. Rather than defining a distinct γδ T cell lineage, KIR expression, therefore, serves as a robust phenotypic marker of advanced effector differentiation, providing a practical framework to study γδ T cell maturation and functional specialization.

This differentiation state is reflected in the distinct phenotypic profiles of KIR^+^ versus KIR^–^ γδ T cells. KIR^+^ γδ T cells are marked by a CD27^–^CD57^+^CX3CR1^+^ profile, indicative of advanced differentiation. In addition, they express elevated levels of diverse NK cell–associated receptors and contain high amounts of granzyme B and perforin, key effectors of cytolytic function. In contrast, KIR^–^ γδ T cells predominantly display a CD27^+^CD57^–^CX3CR1^–^ phenotype, consistent with a naive state. This phenotypic dichotomy was observed across all Vδ1, Vδ2, and Vδ1/2^–^ populations, suggesting that KIR expression marks a common differentiation trajectory for all γδ T cells.

Chronic viral exposure appears to shape this differentiation trajectory. In NK cells, CMV infection typically drives clonal expansion marked by coordinated upregulation of both NKG2C and KIRs (33–36). In contrast, CMV imprinting in γδ T cells is reflected only by elevated KIR expression, with no corresponding increase in NKG2C. Intriguingly, 2 of the 35 CMV^–^ studied donors also harbored a high frequency of KIR^+^ Vδ2 cells, suggesting that factors beyond CMV may contribute to KIR upregulation. While phenotypic differences between KIR^–^ and KIR^+^ γδ T cells were observed across both CMV^–^ and CMV^+^ individuals, these distinctions were substantially more pronounced in CMV^+^ donors, suggesting that chronic viral stimulation amplifies this divergence. These findings parallel reports of expanded, NK-like, KIR-expressing CD8^+^ γδ T cell subsets during chronic infections such as *M. tuberculosis* and other prolonged inflammatory states (20), further supporting the association between KIR expression, chronic immune activation, and the acquisition of a memory-like effector program in human γδ T cells (20).

Of note, although the Vγ9^–^Vδ2^+^ T cell population was more readily detectable in CMV^+^ individuals, consistent with previously reported data (41, 55), it remained at very low frequencies and showed no detectable KIR expression. Overall, this subset represents a negligible fraction of the Vδ2 compartment and is therefore unlikely to meaningfully contribute to the phenotypic differences observed between KIR^+^ and KIR^–^ Vδ2 cells. Accordingly, the Vδ2 population analyzed in this study predominantly reflects Vγ9^+^Vδ2^+^ T cells, supporting the validity of the comparisons presented.

We have shown that KIR expression positively correlates with granzyme B levels and CD57 expression, meaning the more KIRs a γδ T cell expresses, the more cytotoxic and terminally differentiated it tends to be. In NK cells, interactions between inhibitory KIRs and self-MHC molecules establish a consistent, quantifiable relationship between self-recognition and functional potential, a process known as NK cell education (56, 57). A study suggests that inhibitory KIR-ligand interactions remodel the lysosomal compartment, leading to an intracellular build-up of lytic granules and, ultimately, to heightened effector capacity (58). Our data point to an analogous adaptation in γδ T cells. Although we did not directly test for γδ T cell education, flow-cytometry and RNA-seq analyses show that KIR^+^ γδ T cells preferentially express inhibitory receptors, with KIR2DL2/L3 predominating. Most donors in our cohort carry HLA-C1 alleles (C1/C1 or C1/C2), providing the cognate ligand for KIR2DL2 and KIR2DL3. The presence of this ligand-receptor pair offers a mechanistic explanation for the observed association between KIR expression, granzyme B loading, and the terminally differentiated and cytotoxic phenotype of KIR^+^ γδ T cells.

Functionally, as expected, KIR^+^ γδ T cells were marked by significantly higher levels of intracellular IFN-γ, TNF-α, and CCL5 following short-term stimulation, demonstrating both rapid and robust cytokine production. This response was detectable early after stimulation, highlighting the effector potential of this subset. In contrast, KIR^–^ γδ T cells showed a markedly weaker cytokine response under the same conditions. Additionally, KIR^–^ γδ T cells expressed elevated levels of RORγt and produced more IL-17 when cultured under IL-17–polarizing conditions, further highlighting the functional divergence within γδ T cells. Notably, this dichotomy appears specific to γδ T cells, as parallel analyses of NK cells revealed no significant differences in cytokine production between KIR^+^ and KIR^–^ subsets. These findings suggest that the influence of KIR expression on effector function is context dependent and likely shaped by lineage-specific regulatory mechanisms. The observation that ADCC enhancement in KIR^+^ cells was restricted to the Vδ2 subset remains unresolved. Future studies should explore this discrepancy on a larger scale and assess whether prior activation is required to elicit stronger ADCC responses in Vδ2^-^ subsets.

The epigenetic and transcriptional analyses further support these findings. A pairwise comparison of KIR^–^ versus KIR^+^ γδ T cell subsets from 6 individuals revealed that KIR^+^ γδ T cells have increased chromatin accessibility and transcript abundance at key effector loci, including genes encoding a wide range of NK cell receptors as well as core cytotoxic genes such as *GZMB*, *GZMH*, *PRF1*, *IFNG*, and *TBX21*. These regions were transcriptionally active, as confirmed by RNA-seq. Although these molecular features were observed in the absence of stimulation, they anticipated the rapid functional responsiveness observed upon activation, reinforcing the concept of transcriptional and epigenetic “priming” in this subset. This signature closely matches the effector program recently described in adaptive-like Vδ1 T cells (19).

In contrast, KIR^–^ γδ T cells exhibited increased accessibility and expression of genes associated with naive or early memory states, including CD27, CD28, and CCR7, as well as a transcriptional profile consistent with IL-17 production. These cells expressed higher levels of RORC, IL-23R, AREG, IL-17F, CCR6, and CCL20, supporting their identity as tissue-trafficking and IL-17–producing γδ T cells (9–11, 48–51). This molecular signature aligns with the functional data showing enhanced IL-17 production and RORγt expression in KIR^–^ γδ T cells under IL-17–polarizing conditions. IL-17 expression by human γδ T cells remains a topic of active investigation, with relatively few studies to date demonstrating the capacity of peripheral blood γδ T cells to produce this cytokine (10, 48, 59, 60). Many of these studies attribute IL-17 production primarily to the Vδ2 subset (10, 59, 60). However, IL-17 expression has also been reported in other subsets (48). Although our polarization assay revealed consistent differences in detectable IL-17 expression between KIR^–^ and KIR^+^ γδ T cells, we were unable to induce robust cytokine production, despite testing multiple cytokine concentrations and culture durations, as prolonged culture resulted in the loss of KIR^+^ cells.

Together, these findings underscore a clear functional and transcriptional bifurcation within the circulating γδ T cell pool: KIR^–^ cells are biased toward an IL-17–producing and biosynthetically active phenotype, whereas KIR^+^ γδ T cells are transcriptionally and functionally primed for rapid cytotoxic responses.

GSEA further confirmed this division, showing that KIR^+^ γδ T cells are enriched for pathways involved in leukocyte activation, vesicle trafficking, NK-mediated cytotoxicity, and immune effector responses. These features are consistent with a memory-like effector state. Conversely, KIR^–^ γδ T cells showed enrichment for gene sets associated with translation, RNA metabolism, and mitochondrial function, suggestive of a biosynthetic program aligned with ongoing differentiation or proliferation rather than immediate effector function.

In conclusion, our findings identify KIRs as valuable markers of a mature and functionally potent subset of human γδ T cells, with possible implications for immunotherapy. The role of KIR^+^ γδ T cells in infection and cancer, especially in contexts of impaired HLA class I expression, remains largely unexplored. Many viruses and tumors evade immunity by downregulating HLA class I, a strategy observed in up to 90% of cancers (61). While “missing-self” recognition by KIR^+^ NK cells is well established, the contribution of KIR^+^ γδ T cells in this setting is only beginning to emerge. A key example comes from mismatch repair-deficient colorectal cancers, where loss of β2-microglobulin leads to HLA class I deficiency. In this context, KIR^+^ γδ T cells infiltrate tumors and are associated with better responses to anti–PD-1 therapy and improved prognosis (31). Our data support the potential of KIR^+^ γδ T cells as a promising cell population for adoptive immunotherapy, including CAR engineering or combination with immune engagers. Their memory-like phenotype further suggests capacity for in vivo persistence, long-term immune surveillance, and durable antitumor immunity.

Future studies should clarify how KIR^+^ γδ T cells develop, how KIRs shape their education and functional tuning, and what roles these cells play in infection, cancer, and immune homeostasis. Defining the ligand specificities and signaling pathways downstream of KIRs will also be key to understanding how these receptors fit into the broader landscape of γδ T cell activation and adaptation.

## Methods

### Sex as a biological variable.

Both male and female participants were included and analyzed together, as no sex-related differences were observed. Our protocol allowed enrollment regardless of sex and ethnicity, and recruitment reflected this principle.

### Cells and cell culture.

Peripheral blood samples from healthy adult volunteers were first centrifuged at 750 × *g* for 10 min to separate the plasma from cellular components. PBMCs were then isolated from the remaining heparinized blood using Ficoll Paque Plus (Sigma) density gradient centrifugation, following the manufacturer’s instructions. Isolated PBMCs were cryopreserved in fetal bovine serum (FBS; Gemini) supplemented with 10% dimethyl sulfoxide (DMSO; Sigma-Aldrich). Both plasma and PBMCs were anonymized and biobanked for subsequent analyses.

CMV serostatus was determined for each donor using thawed plasma samples and a CMV IgG ELISA kit (Calbiotech), following the manufacturer’s instructions. Cryopreserved PBMCs were thawed, suspended in RPMI 1640 medium (Corning Cellgro), and centrifuged for 5 minutes at 500 × *g*. Cell pellets were then resuspended in RPMI 1640 supplemented with 10% heat-inactivated FBS, 2 mM L-glutamine, and 100 IU/100 μg/mL streptomycin/penicillin and incubated overnight at 37°C to allow recovery from freeze-thaw stress prior to in vitro experimentation. αβ T cells were subsequently depleted from total PBMCs using the EasySep Human Alpha/Beta T Cell Depletion Kit (STEMCELL Technologies), following the manufacturer’s instructions. Raji (ATCC CCL86), a Burkitt lymphoma cell line, was cultured in RPMI-1640 with 10% heat-inactivated FBS, 100 IU/100 μg/mL penicillin/streptomycin, and 2 mM L-glutamine.

### Functional assays.

To evaluate the capacity of cells to produce cytokines, αβ T-depleted PBMCs were stimulated with PMA/Ionomycin (BioLegend), according to the manufacturer’s recommendations. Cells were cultured for 4 hours in a 96-well plate in RPMI 1640 containing 10% heat-inactivated FBS, 2 mM L-glutamine, 100 IU/100 μg/mL streptomycin/penicillin, and 200 IU/mL recombinant human IL-2. Brefeldin A (BioLegend) was added to the cultures for the last 3 hours. Cells were then stained for flow cytometry analysis, which measured the frequency of γδ T cells and NK cells expressing IFN-γ, TNF-α, and CCL5. For ADCC assays, Raji cells (1 × 10^6^ cells/mL) were precoated with Rituximab (Genentech) at 10 μg/mL for 30 minutes, before being washed with RPMI-1640. These target cells were coincubated with αβ T cell–depleted PBMCs at an effector/target ratio of 5:1 for 5 hours. Brefeldin A was added for the last 4 hours. Cells were subsequently stained and analyzed by flow cytometry to assess γδ T cells expressing CD107a and IFN-γ.

To assess RORγt expression and IL-17 production, γδ T cells were polarized into IL-17–producing cells by culturing for 4 days in RPMI medium supplemented with 10% FBS, 15 ng/mL IL-1β (Miltenyi Biotec), 30 ng/mL IL-6, 15 ng/mL IL-23, and 2 ng/mL TGF-β (Thermo Fisher Scientific). After polarization, cells were washed and restimulated with PMA/Ionomycin in the presence of Brefeldin A for 4 hours at 37°C. Flow cytometry was then used to measure the frequency of γδ T cells expressing RORγt and IL-17. In some experiments, γδ T cells were cultured for 10 days with irradiated allogeneic PBMCs in the same IL-17–polarizing conditions, and KIR expression was assessed on Day 10.

### Flow cytometry.

αβ T cell–depleted PBMCs were stained with either Ghost Dye Red 710 (Sigma-Aldrich) or the Zombie Aqua Fixable Viability Kit (BioLegend) to discriminate live from dead cells. Depending on the experiment, cells were surface-stained with antibodies listed in Supplemental Table 1. Fc receptor blocking solution was applied before antibody staining in all analyses to minimize nonspecific binding.

In most instances, anti-KIR2D and anti-KIR3DL1/S1 antibodies were conjugated to the same fluorochrome, allowing the combined identification of KIR2D^+^KIR3D^+^ γδ T cells, referred to as KIR^+^ γδ T cells throughout the manuscript. Supplemental Figure 7 details the gating strategy used in our analyses. It also includes the appropriate controls validating the simultaneous staining of KIR2D and KIR3D antibodies conjugated to the same fluorochrome, allowing visualization of the individual staining and supporting the validity of the technical approach. Notably, the proportions of KIR2D^+^ and KIR3D^+^ cells observed in the individual staining closely match the proportion of KIR2D^+^KIR3D^+^ cells detected in the combined staining.

Cells were fixed and permeabilized using either the Intracellular Fixation & Permeabilization Buffer Set or the Transcription Factor Buffer Set in the case of RORγt (Thermo Fisher Scientific), following the manufacturer’s instructions. After staining with antibodies targeting intracellular markers listed in Supplemental Table 1, cells were resuspended in PBS, and data were acquired on a Northern Lights spectral flow cytometer (Cytek) and analyzed using FlowJo software (BD).

### ATAC-seq.

Samples were obtained from CMV^+^ donors, as these individuals typically harbor sufficient frequencies of KIR^+^ γδ T cells. After depletion of αβ T cells, PBMCs were stained with antibodies against CD3, KIR2D, and KIR3DL1/S1, as well as the Zombie Aqua Viability Dye, as previously described. Subsequently, 50,000 KIR^–^ and 50,000 KIR^+^ γδ T cells were sorted using the Sony SH800 cell sorter (Flow Cytometry and Virometry Facility, University of Ottawa). ATAC-seq was performed as previously described (47). The cells were pelleted and processed using the OMNI-ATAC protocol (46), employing home-made Tn5 transposase prepared as reported (62). Libraries were dual indexed and size selected to retain fragments between 200 and 1,000 base pairs. Sequencing was performed at The Centre for Applied Genomics, Toronto. Paired-end sequencing was performed for a total of 300 cycles. Sequencing data are available at NCBI GEO under accession no. GSE301798.

Sequencing data were analyzed using a pipeline previously described (63), with 1 modification: alignment with STAR allowed mapping at up to 50 positions to ensure detection of KIR loci on alternate haplotype chromosomes. Differential DNA accessibility analysis was performed in R/Bioconductor (https://www.r-project.org/) using a sliding window approach based on the csaw package (64, 65), with windows of 50 bp shifted by 50 bp quantifying the transposase insertion sites. Library sizes were normalized using the TMM method. Unwanted variance was removed using RUVseq, employing the RUVr algorithm and k = 1 setting (66, 67). Differential testing was performed using a paired design testing the influence of the KIR status (~Patient_number + KIR_status + W_1) using KIR^-^ as the baseline, where W_1 is the factor of unwanted variation from RUVr. Differentially accessible regions (DARs) were identified as windows with a Benjamini-Hochberg-adjusted *P* value (FDR) lower than 0.05 and absolute value of log_2_FC greater than 1; significant windows that were immediately adjacent were merged into “regions.” Heatmaps were created with the ComplexHeatmaps package (68), based on scaled and centered normalized insertion counts across regions in each sample, after applying RUVseq. DARs were assigned to genes using the package ChIPseeker (69), assigning each DAR to the gene with the nearest TSS.

### RNA-seq.

In total, 50,000 KIR^–^ and 50,000 KIR^+^ γδ T cells from CMV^+^ individuals were sorted as described above for ATAC-seq. Total RNA was extracted from the sorted cells using the RNAeasy Plus Micro kit (Qiagen). RNA-seq was performed at The Centre for Applied Genomics. Briefly, RNA libraries were prepared using the NEBNext Ultra II Directional RNA Library Prep protocol with 50 ng of total RNA. Poly-A mRNA was enriched, fragmented, and converted into double-stranded cDNA. After end repair and adapter ligation, libraries were amplified with barcoded adapters for multiplexing. Library size and quality were assessed using a Bioanalyzer, and concentrations were determined by qPCR. Libraries were pooled in equimolar amounts and sequenced on the Illumina NovaSeq X platform to generate 150 bp paired-end reads. Sequencing data are available at NCBI GEO, under accession no. GSE301799.

Reads were processed essentially as previously described (47). Read pairs were summarized to genes using Subread featureCounts function (70). The read count matrix was filtered to retain only genes with counts higher than 10 in at least 2 samples and then analyzed in R/Bioconductor with the edgeR package glmQLFTest (71) to identify differentially expressed genes (DEGs). As for ATAC-seq, unwanted variance was removed using the RUVr algorithm and k = 1 option and a paired (by donor) design was used to identify the effect of KIR status on gene expression.GSEA was performed using the clusterProfiler package with the following parameters: minGSSize = 10 (minimum gene set size), maxGSSize = 1000 (maximum gene set size), pvalueCutoff = 1.0 (nominal P-value threshold for reporting results), pAdjustMethod = “BH” (adjustment of P-values using the Benjamini-Hochberg method), and eps = 0 (threshold below which P-values are calculated instead of estimated). Ranking of genes was based on the significance of the differential expression test result, using –log(*P* value) × sign(log_2_FC) as the metric. Gene sets from the MSigDB database (73) were obtained using the ExperimentHub and msigdb packages; only sets from the “Hallmark,” “Reactome,” and “Gene Ontology” sections were retained for testing. Gene sets with FDR < 0.05 and an absolute value of normalized enrichment score (NES) greater than 1.5 were considered significant. After manual inspection of all significant results, specific sets were retained to be part of the final figure.

### HLA-C group 1 and 2 KIR ligand genotyping.

The presence or absence of HLA-C molecules bearing C1 or C2 epitopes, recognized by lineage III KIRs (32, 74), was determined using a PCR sequence-specific primer (PCR-SSP) method, as previously described (75).

### Statistics.

Statistical analyses were performed using Prism software. Paired 2-tailed *t* tests were used to compare KIR^–^ and KIR^+^ γδ T cells, while unpaired 2-tailed *t* tests were applied for comparisons between CMV^–^ and CMV^+^ individuals. *P* < 0.05 was considered statistically significant. For the bioinformatic analysis of the sequencing data, multiple hypothesis correction was performed using the Benjamini-Hochberg algorithm to produce false discovery rate values (FDR).

### Study approval.

Blood samples were obtained from healthy adult volunteers who provided written informed consent, as per study approval by the Ottawa Hospital Research Institute Ethics Board (OHRI REB 2005256-01H).

### Data availability.

Sequencing data are available at NCBI GEO under accession GSE301798 and GSE301799, for ATAC-seq and RNA-seq, respectively. Supporting Data Values are included, containing all data points shown in the graphs, with separate tabs for each figure panel.

## Author contributions

MR purified immune cells, conducted part of the immune profiling and functional assays, extracted RNA samples, prepared the ATAC-seq libraries, genotyped samples for the presence or absence of HLA-C1 or C2 alleles. YA purified immune cells, performed functional assays, contributed to figure preparation, revised the manuscript by performing additional immune profiling, analyzing the data, designing supplemental figures, and contributing to the manuscript revision process. ML contributed by biobanking immune cells and assessing the CMV serological status of the blood donors. JBA supervised blood donor collection. AB oversaw ATAC library preparation and conducted sequencing analyses for both ATAC-seq and RNA-seq. ZD conceived the project, analyzed data and wrote the manuscript. The co–first authors contributed equally overall; however, the order reflects relatively greater involvement in experimental execution and data analysis by the first-listed author.

## Conflict of interest

The authors have declared that no conflict of interest exists.

## Funding support

- Canada Research Chairs (CRC; 202200508) to ZD
- Canada Foundation for Innovation (CFI; 43550) to ZD
- Natural Sciences and Engineering Research Council (NSERC; DGECR-2024-00242) to ZD
- Cancer Research Society (CRS; OG202489) to ZD
- Canadian Institutes of Health Research (CIHR; PJ4-196041) to ZD and CIHR (PJT-183839) to AB

## Supplementary Material

Supplemental data

Supporting data values

## Version 1 04/27/2026

In-Press Preview

## Version 2 06/08/2026

Electronic publication
