# Supplementary material for: Killer-cell immunoglobulin-like receptors define a potent effector program in human γδ T cells
Source: JCI Insight. 2026 Apr 23;11(11):e201160. doi: 10.1172/jci.insight.201160 (PMC13313560; doi:10.1172/jci.insight.201160)
Supplement: Supplemental data [file jciinsight-11-201160-s012.pdf]

Figure S1

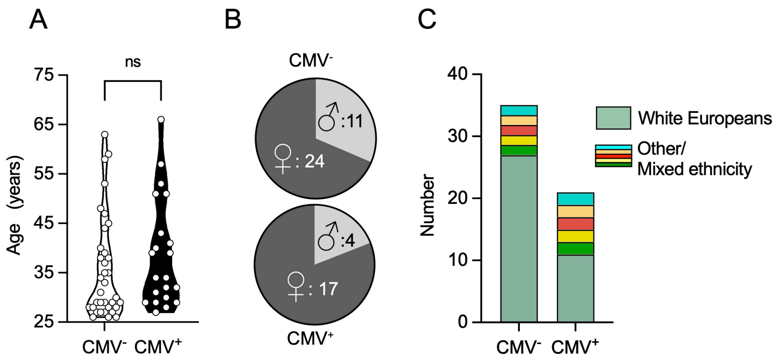

A
ns
Age (years)
75
65
55
45
35
25
CMV⁻ CMV⁺
B
CMV⁻
♂:11
♀: 24
♂:4
♀: 17
CMV⁺
C
40
30
20
10
0
Number
White Europeans
Other/
Mixed ethnicity
CMV⁻ CMV⁺

Figure S1. Cohort of healthy adult blood donors.

A. Violin plots compare the ages of healthy adult participants in the CMV^{-} and CMV^{+} groups. **B.** Pie charts illustrate the sex distribution (♀/♂) within the CMV^{-} and CMV^{+} groups. **C.** Bar graphs show the distribution of self-reported ethnic origins across the two groups.

The cohort includes 56 individuals in total: 35 CMV^{-} and 21 CMV^{+}.

Statistical significance of the age difference between CMV^{-} and CMV^{+} was assessed using the paired two-tailed t test (ns = nonsignificant).

Figure S2

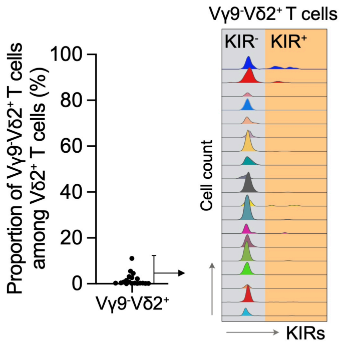

Proportion of Vγ9⁻Vδ2⁺ T cells among Vδ2⁺ T cells (%)
100
80
60
40
20
0
Vγ9⁻Vδ2⁺
Vγ9⁻Vδ2⁺ T cells
KIR⁻
KIR⁺
Cell count
KIRs

Figure S2. Vγ9$^-$Vδ2$^+$ T cells are a minor population in CMV$^+$ healthy individuals and exhibit minimal KIR expression.

A. Graph shows the proportion of Vγ9$^-$Vδ2$^+$ T cells within the Vδ2 T cell population in CMV$^+$ individuals.

B. Histogram overlay depicts KIR expression on Vγ9$^-$Vδ2$^+$ T cells from each CMV$^+$ individual.

Figure S3

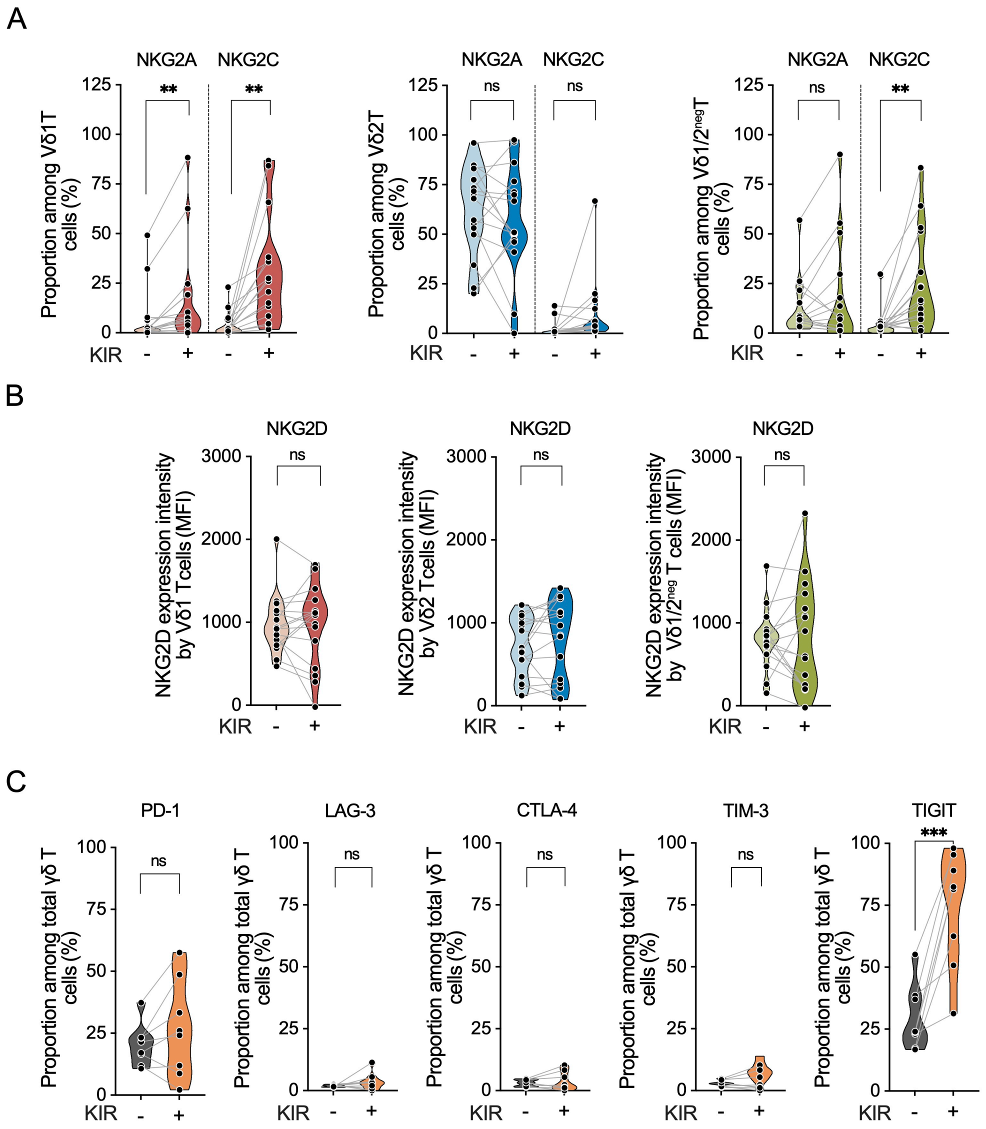
A
NKG2A
NKG2C
Proportion among Vδ1T cells (%)
Proportion among Vδ2T cells (%)
Proportion among Vδ1/2negT cells (%)
125
100
75
50
25
0
**
ns
KIR
-
+
B
NKG2D
NKG2D expression intensity by Vδ1 Tcells (MFI)
NKG2D expression intensity by Vδ2 Tcells (MFI)
NKG2D expression intensity by Vδ1/2neg T cells (MFI)
3000
2000
1000
C
PD-1
LAG-3
CTLA-4
TIM-3
TIGIT
Proportion among total γδ T cells (%)
***

Figure S3. Differential expression of additional NK cell markers and checkpoint inhibitors.

A. Violin plots compare the proportions of KIR^{-} and KIR^{+} cells expressing the indicated markers within Vδ1 (pink, left panel), Vδ2 (blue, middle panel) and $V\delta 1/2^{neg}$ (green, right panel) T cells.

B. Violin plots compare the expression intensity of NKG2D on KIR^{-} and KIR^{+} cells within Vδ1 (pink, left panel), Vδ2 (blue, middle panel) and $V\delta 1/2^{neg}$ (green, right panel) T cell subsets.

C. Violin plots compare the proportions of KIR^{-} and KIR^{+} cells expressing the indicated checkpoint inhibitors within total γδ T cells.

Each dot represents an individual donor (N=16, **A**, **B**; N=8, **C**). Statistical significance of the difference between KIR^{-} and KIR^{+} cells was assessed using the paired two-tailed t test (**= $p<0.005$, ns = nonsignificant).

Figure S4

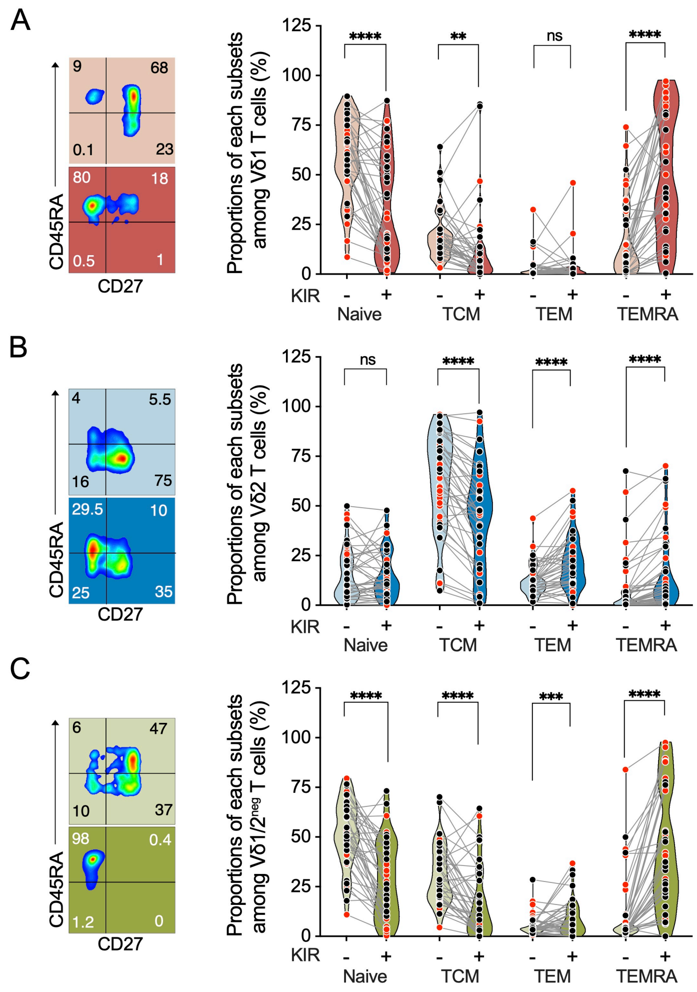
A
B
C
CD45RA
CD27
Proportions of each subsets among Vδ1 T cells (%)
Proportions of each subsets among Vδ2 T cells (%)
Proportions of each subsets among Vδ1/2neg T cells (%)
KIR
Naive
TCM
TEM
TEMRA
****
**
ns
***
9
68
0.1
23
80
18
0.5
1
4
5.5
16
75
29.5
10
25
35
6
47
10
37
98
0.4
1.2
0
125
100
75
50
25
0

Figure S4. All KIR$^+$ γδ T cell subsets predominantly exhibit a TEMRA-like phenotype.

A-C. Representative pseudocolor plots (left panels) and corresponding violin plots (right panels) illustrate the distribution of CD27/CD45RA-defined subsets among KIR$^−$ (lighter tones) and KIR$^+$ γδ T cells (darker tones). These subsets include naïve, central memory (TCM), effector memory (TEM), and terminally differentiated effector memory (TEMRA) populations across Vδ1 (**A**), Vδ2 (**B**), and Vδ1/2^{neg} (**C**) subsets. Each dot represents an individual donor (n = 40). Samples from CMV$^+$ individuals are shown in red symbols. Statistical significance of the difference between KIR$^-$ and KIR$^+$ cells was assessed using the paired two-tailed t test (**p≤0.005,***= p≤0.001, ****p≤0.0001, ns = nonsignificant).

Figure S5

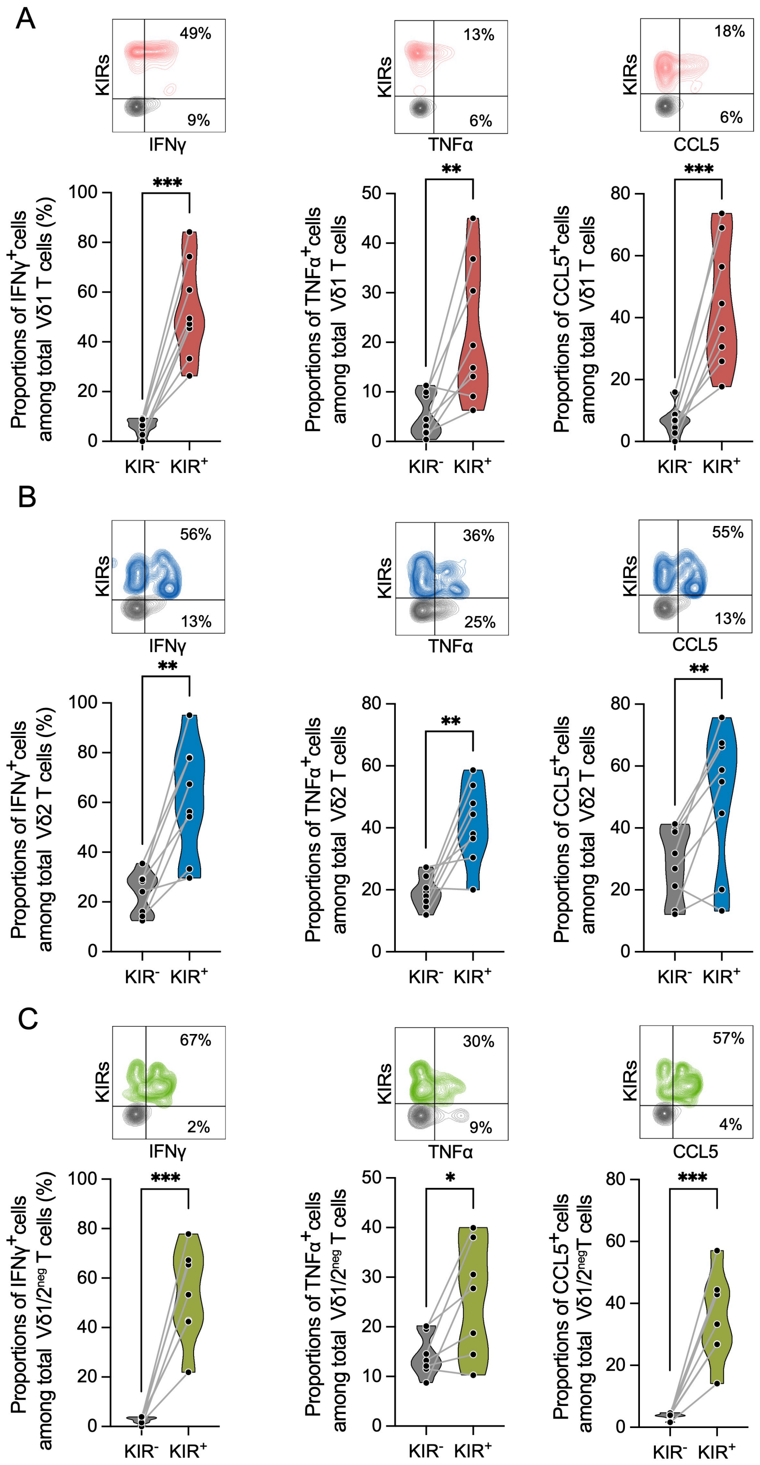
A
KIRs
IFNγ
49%
9%
TNFα
13%
6%
CCL5
18%
6%
Proportions of IFNγ+cells among total Vδ1 T cells (%)
Proportions of TNFα+cells among total Vδ1 T cells
Proportions of CCL5+cells among total Vδ1 T cells
***
**
***
KIR- KIR+
B
56%
13%
36%
25%
55%
13%
Proportions of IFNγ+cells among total Vδ2 T cells (%)
Proportions of TNFα+cells among total Vδ2 T cells
Proportions of CCL5+cells among total Vδ2 T cells
**
**
**
C
67%
2%
30%
9%
57%
4%
Proportions of IFNγ+cells among total Vδ1/2neg T cells (%)
Proportions of TNFα+cells among total Vδ1/2neg T cells
Proportions of CCL5+cells among total Vδ1/2negT cells
***
*
***

Figure S5: KIR$^+$ γδ T cells exhibit elevated levels of cytokine production following activation across γδ T cell subsets.

A–C. Representative overlaid contour plots (top panels) and violin plots (bottom panels) compare proportions of KIR$^-$ (grey) and KIR$^+$ (pink for Vδ1 (**A**), blue for Vδ2 (**B**), green for Vδ1/2^{neg} (**C**)) T cells producing IFNγ, TNFα and CCL5, after 4 hours of PMA/ionomycin stimulation.

Each dot represents an individual donor (N=8 for Vδ1 and Vδ2; N=7 for Vδ1/2^{neg}, as one donor lacks KIR expression in this subset). Statistical significance of the difference between KIR$^-$ and KIR$^+$ subsets was assessed using the paired two-tailed t test (*p=0.02, **p≤0.009, ***p≤0.0006, ****p<0.0001).

Figure S6

A

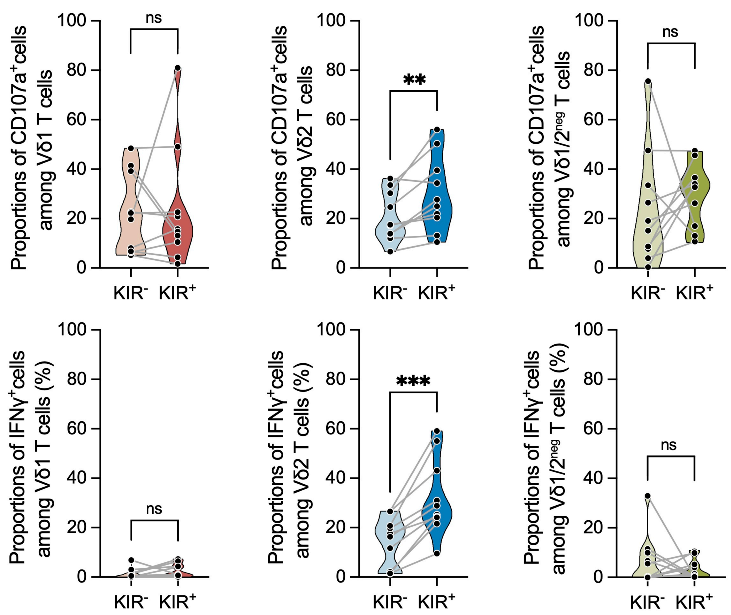

Proportions of CD107a+cells among Vδ1 T cells
Proportions of CD107a+cells among Vδ2 T cells
Proportions of CD107a+cells among Vδ1/2neg T cells
Proportions of IFNγ+cells among Vδ1 T cells (%)
Proportions of IFNγ+cells among Vδ2 T cells (%)
Proportions of IFNγ+cells among Vδ1/2neg T cells (%)
ns
**
***
KIR- KIR+

B

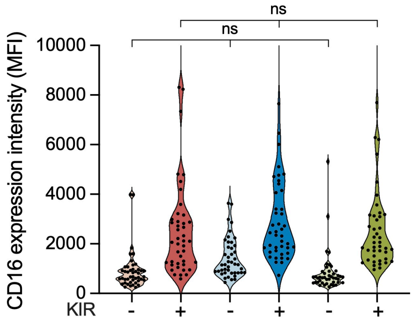

CD16 expression intensity (MFI)
ns
ns
KIR
- + - + - +

Figure S6: KIR expression is associated with enhanced ADCC in Vδ2 T cells but not in other γδ T cell subsets.

A. Violin plots illustrate degranulation (top panels) and IFNγ production (bottom panel) in KIR^{-} and KIR^{+} populations of Vδ1 (pink), Vδ2 (blue) and Vδ1/2^{neg} (green) γδ T cells following an ADCC assay. Cells were co-cultured for 5 hours with Rituximab-coated Raji cells. Data represent the percentage of $CD107a^{+}$ or $IFN\gamma^{+}$ cells within each indicated subset. Each dot represents an individual donor (N=10).

B. Violin plots depict CD16 surface expression levels, quantified as median fluorescence intensity (MFI), in the same γδ T cell subsets, color-coded as in panel A and stratified by KIR expression. Each dot represents an individual donor (N=44).

Statistical significance between KIR^{-} and KIR^{+} populations within each subset was assessed using the paired two-tailed t test (**p≤0.007, ***p≤0.0002, ns = nonsignificant).

Figure S7

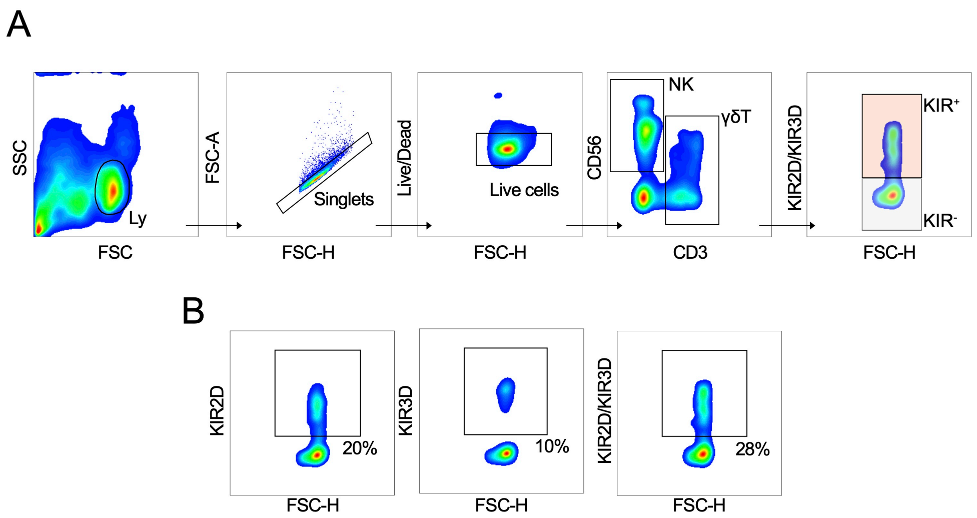
A
SSC
FSC
Ly
FSC-A
FSC-H
Singlets
Live/Dead
FSC-H
Live cells
CD56
CD3
NK
γδT
KIR2D/KIR3D
FSC-H
KIR+
KIR-
B
KIR2D
FSC-H
20%
KIR3D
FSC-H
10%
KIR2D/KIR3D
FSC-H
28%

Figure S7: Gating strategy.

A. Representative pseudocolor plots illustrate sequential gating strategy for KIR^{-} and KIR^{+} populations within αβ T cell-depleted PBMCs. KIR^{-} and KIR^{+} populations were defined using KIR2D/KIR3D antibodies conjugated to the same fluorochrome.

B. Representative pseudocolor plots showing single stain controls and the combined staining.

Supplementary Table 1: List of monoclonal antibodies used for flow cytometry analysis

Specificity	Clone	Vendor; Catalog number
CD3	OKT3	BioLegend; 317323
CD56	5.1H11	BioLegend; 362549
CD14	M5E2	BioLegend; 301833
CD19	HIB19	BioLegend; 302227
TCR αβ	IP26	BioLegend; 306705
TCR Vγ9	B3	BioLegend; 331328
TCR Vδ1	REA173	Miltenyi Biotec; 130-120-583
TCR Vδ2	123R3	Miltenyi Biotec; 130-125-878
CD27	L128	BD Biosciences; 563816
CD45RA	HI100	BioLegend; 304141
CD244	C1.7	BioLegend; 329519
CD57	REA769	Miltenyi Biotec; 130-111-968
CD16	REA423	Miltenyi Biotec; 130-113-395
LILRB1	HP-F1	Thermo Fisher Scientific; 367-5129-41
NKG2A	REA1161	Miltenyi Biotec; 130-120-377
NKG2C	REA205	Miltenyi Biotec; 130-119-776
NKG2D	1D11	BioLegend; 285147
KIR2D	REA1042	Miltenyi Biotec; 130-117-479
KIR2DL1	REA284	Miltenyi Biotec; 130-118-484
KIR2DL1/S1	REA1010/11PB6	Miltenyi Biotec; 130-118-973
KIR2DL2/L3	REA1006/DX27	Miltenyi Biotec; 130-116-954
KIR2DL2/L3/S2	DX27	BioLegend; 312623
KIR2DS4	REA860	Miltenyi Biotec; 130-114-773
KIR3DL1/S1	REA168	Miltenyi Biotec; 130-128-302
KIR3DL1	DX9	BioLegend; 312729
PD-1	A17188B	BioLegend; 621619
TIM-3	F38-2E2	BioLegend; 345008
CTLA-4	L3D10	BioLegend; 349905
TIGIT	A15153G	BioLegend; 372704
LAG-3	3DS223H	Thermo Fisher Scientific; 17-2239-42
CD107a	LAMP-1	BioLegend; 328656
Granzyme B	REA226	Miltenyi Biotec; 130-116-486
Perforin	dG9	BioLegend; 308129
IFNγ	REA600	Miltenyi Biotec; 130-132-901
TNFα	REA656	Miltenyi Biotec; 130-127-550
CCL5	VL1	BioLegend; 515507
IL-17A	BL168	BioLegend; 512305
RORγt	REA278	Miltenyi Biotec; 130-124-035

Surface markers	Intracellular markers
